# In Vitro Assessment of Enteric Methane Emission Potential of Whole-Plant Barley, Oat, Triticale and Wheat

**DOI:** 10.3390/ani11020450

**Published:** 2021-02-09

**Authors:** Isaac A. Aboagye, Christine L. Rosser, Vern S. Baron, Karen A. Beauchemin

**Affiliations:** 1 Lethbridge Research and Development Centre, Agriculture and Agri-Food Canada, 5403 1st Avenue South, Lethbridge, AB T1J 4B1, Canada; isaac.aboagye@umanitoba.ca (I.A.A.); christine.rosser@canada.ca (C.L.R.); 2 Lacombe Research and Development Centre, Agriculture and Agri-Food Canada, Lacombe, AB T4L 1W1, Canada; vern.baron@canada.ca

**Keywords:** barley, oat, wheat, triticale, nutrient degradability, methane emission

## Abstract

**Simple Summary:**

There is an increasing interest in finding effective but economical strategies for mitigating enteric methane (CH_4_) emissions from ruminants. Small-grain cereal forages including barley, oat, triticale, and wheat, unlike maize, are widely grown in temperate locations and may be economical to use for ruminant production. However, the starch and fiber composition and concentrations of whole-plant cereal forages affect rumen degradability, and hence may cause differences in the CH_4_ production potential among these forages. Therefore, the objective of this study was to determine the enteric CH_4_ emission potential of various whole-plant cereals and evaluate whether the variability in emissions could be explained by variations in nutrient profiles, degradability, and rumen fermentation characteristics. The results indicate that feeding whole-plant oat forage to ruminants may decrease CH_4_ emissions but adversely affect animal performance due to lower degradability, whereas barley forage may ameliorate emissions without negative effects on animal performance.

**Abstract:**

The study determined in vitro enteric methane (CH_4_) emission potential of whole-plant cereal (WPC) forages in relationship to nutrient composition, degradability, and rumen fermentation. Two varieties of each WPC (barley, oat, triticale, and wheat) were harvested from two field replications in each of two locations in central Alberta, Canada, and an in vitro batch culture technique was used to characterize gas production (GP), fermentation, and degradability. Starch concentration (g/kg dry matter (DM)) was least (*p* < 0.001) for oat (147), greatest for wheat (274) and barley (229), and intermediate for triticale (194). The aNDF concentration was greater for oat versus the other cereals (531 vs. 421 g/kg DM, *p* < 0.01). The 48 h DM and aNDF degradabilities (DMD and aNDFD) differed (*p* < 0.001) among the WPCs. The DMD was greatest for barley, intermediate for wheat and triticale, and least for oat (719, 677, 663, and 566 g/kg DM, respectively). Cumulative CH_4_ production (MP; mL) from 12 h to 48 h of incubation was less (*p* < 0.001) for oat than the other cereals, reflecting its lower DMD. However, CH_4_ yield (MY; mg of CH_4_/g DM degraded) of barley and oat grown at one location was less than that of wheat and triticale (28 vs. 31 mg CH_4_/g DM degraded). Chemical composition failed to explain variation in MY (*p* = 0.35), but it explained 45% of the variation in MP (*p* = 0.02). Variation in the CH_4_ emission potential of WPC was attributed to differences in DMD, aNDFD, and fermentation end-products (R^2^ ≥ 0.88; *p* < 001). The results indicate that feeding whole-plant oat forage to ruminants may decrease CH_4_ emissions, but animal performance may also be negatively affected due to lower degradability, whereas barley forage may ameliorate emissions without negative effects on animal performance.

## 1. Introduction

Enteric methane (CH_4_) emissions from ruminants are estimated at 1.6 to 2.7 gigatons of carbon dioxide equivalent (CO_2_e) per year, accounting for 3% to 5% of the 49 Gt total CO_2_e produced globally by all sectors [1]. Methane is a potent greenhouse gas and contributes to climate change [2], and thus there is increasing emphasis on reducing enteric CH_4_ emissions from ruminants. Ruminants consume forage-based diets, and improving the nutritional quality of forages enhances feed conversion efficiency and animal performance. However, CH_4_ production from animals may also increase due to greater feed intake and ruminal degradation of high-quality forages. However, CH_4_ produced per unit of animal product (i.e., a measure of intensity) typically decreases with improved animal performance (growing animals reach market weight sooner so less feed is required; fewer lactating dairy cows are needed to produce a given volume of milk) [3].

Grasslands have notable environmental benefits such as carbon storage [4]; however, low-quality forages are estimated to account for 75% of global ruminant CH_4_ emissions [5]. Using a batch culture approach, Macheboeuf et al. [6] showed that methane yield (MY; CH_4_ per unit of organic matter degraded) of forages is extremely variable; some forages produced 80% greater MY than perennial ryegrass. The large variability in MY of forages may be related to their chemical composition (starch and fiber concentrations) and rumen degradability. Greater starch and lower fiber concentrations favor the production of propionate during ruminal fermentation, providing an alternative hydrogen sink to CH_4_ [7]. Therefore, the use of maize and cereal forages, which contain high concentrations of starch and have relatively high digestibility, may help ameliorate enteric CH_4_ emissions from forage-fed ruminants.

Maize is a warm-season crop and not agronomically suitable in many locations. In contrast, small-grain cereal forages (barley, oat, triticale, and wheat) are widely grown in temperate locations. Cereal forages vary in starch and fiber concentrations and rumen degradabilities ([8,9,10], and thus CH_4_ emission would be expected to vary accordingly). However, few studies have examined the variability of CH_4_ emissions among cereal crops when harvested as forages, although several studies have reported CH_4_ emissions for cereals harvested and fed as grains. Replacing barley grain with wheat grain in the diet of dairy cows resulted in 73% to 78% less CH_4_ (g/kg DM intake and g/day; [11]). An in vitro study reported no difference in CH_4_ production (MP; mL) between a diet containing oat grain compared with barley grain, although CH_4_ adjusted for intake (g/kg DM intake) was less for the oat diet [12]. However, comparisons of the CH_4_ potential of cereal grains may not reflect that of whole-plant cereals (WPC) that contain high concentrations of fibrous components (stem, leaves, and hulls).

Little is known about the relationships between enteric CH_4_ emissions (MP and MY) of WPC species (barley, oat, triticale, and wheat) and nutrient composition, degradability and fermentation profile. Thus, the objective of this study was to determine the potential enteric CH_4_ emission of various WPCs and evaluate whether the variability in emissions could be explained by variations in nutrient profiles, degradability, and rumen fermentation characteristics. We hypothesized that WPCs that produce less CH_4_ would also be less degradable, but that adjusting CH_4_ production to account for rumen degradability (i.e., MY) would not eliminate differences among WPCs because forages with greater starch concentration would favor propionate concentration, resulting in less MY.

## 2. Materials and Methods

### 2.1. Whole-Plant Small-Grain Cereal Crops

Four WPC crops (barley, oat, triticale, and wheat) were grown in 2018 at two locations (A = Agriculture and Agri-Food Canada; B = Alberta Agriculture) near Lacombe, Alberta (52°28′ N, 113°45′ W), Canada on an Orthic Black Chernozemic “Penhold” silt loam soil with two field plots (replications) per location.

At each location, two varieties were grown for barley (CDC Cowboy, Champion), oat (AC Mustang, CDC Baler), triticale (Bunker, Sunray), and wheat (AAC Awesome, GP220). The varieties, which are usually grown for silage or greenfeed (fed fresh or dried and preserved as hay) in Alberta, were chosen based on preliminary screening to provide a range in chemical composition and degradability within the cereal crops.

Crop treatments were established in 5.1 m^2^ plots. Seeding occurred May 8 at a rate of 300 seeds m^−2^ with an eight-run shop-fabricated three-point-hitch cone seeder with double disk openers and following packing wheels. Row spacing was 15 cm. Prior to seeding, fertilizers were broadcast to supply 11-55-66 and 7-28-33 kg ha^−1^ of N, P_2_O_5_, and K_2_O at locations A and B, respectively. The soil test available N was in excess of 75 kg ha^−1^, as the experimental areas had been summer-fallowed the previous year. Post-emergent herbicides were applied as recommended for cereal crops [13].

Each plot was rated for developmental stage using the Tottman et al. [14] development scale. Barley, wheat, and triticale harvest occurred as close as practically possible to the soft-dough stage (stage 85) at both locations. Oat was harvested at the medium milk stage (stage 75). Barley was harvested between July 26 and 30; oat was harvested on August 2, wheat on August 4, and triticale on August 7. A 2.81 m^2^ area (eight rows) was harvested from each plot using a Hege model 212 forage harvester (Wintersteiger Inc., Saskatoon, SK, Canada) equipped with a six-knife cutting drum and set to cut 5 cm above ground. A 5-kg subsample of each WPC was obtained for each location and field replication (4 WPCs, 2 varieties, 2 locations, 2 field replications, for a total of 32 samples). The samples were dried for at least 72 h at 55 °C, ground through a 4-mm sieve (standard model 4 Wiley Mill; Arthur Thomas Co., Philadelphia, PA, USA), reground through a 1-mm sieve, and retained for chemical analysis and use in an in vitro batch culture study.

### 2.2. Chemical Analyses

The chemical analysis was conducted in duplicate for DM, ash, crude protein (CP), starch, and neutral detergent fiber including ash (aNDF). Analytical DM and ash concentrations were determined using method 930.15 [15]. Neutral detergent fiber concentration (method 2002.04; [15]) including ash (aNDF) was determined using an Ankom 200^®^ system (Ankom Technology Corporation, Fairport, NY, USA) with heat-stable α-amylase and sodium sulfite used in the assay. Ball-ground (Mixer Mill MM2000; Retsch, Haan, Germany) samples were analyzed for CP (nitrogen × 6.25) and starch concentrations. Nitrogen in CP was analyzed by flash combustion with gas chromatography and thermal-conductivity detection (Nitrogen Analyzer 1500 series; Carlo Erba Instruments, Milan, Italy). Starch concentration was determined as described by Koenig et al. [16] with enzymatic hydrolysis of α-linked glucose polymers.

### 2.3. In Vitro Batch Culture Study

The animal handling and care procedures for this study were approved by the Animal Care Committee of Lethbridge Research and Development Centre and followed the guidelines of the Canadian Council of Animal Care [17]. Three mature, previously cannulated crossbred beef cattle were used to provide pooled rumen inoculum for the batch culture study. The animals were fed a diet containing mainly whole-crop barley silage offered once daily.

The study was a completely randomized design with two in vitro runs conducted sequentially using the batch culture technique as described by Aboagye et al. [18]. Within each run, the ground WPC varieties from both locations and field plots (32 samples) were replicated three times. Gas production (GP) and MP (mL) were measured at specific times throughout the incubation (3, 6, 12, 18, 24, 36, and 48 h), while fermentation characteristics, DMD, and aNDFD were measured after 48 h of incubation.

Acetone-washed ANKOM F57 filter bags (50 μm pore size; Ankom Technology Corp., Macedon, NY, USA), each containing weighed sample (0.7 ± 0.01 g) of ground WPC, were heat-sealed and placed separately into 125 mL serum vials. Before the morning feeding, rumen contents from various locations within the rumen of the three animals were collected, pooled across animals, squeezed through two layers of cheesecloth to collect fluid, and immediately transported to the laboratory in an insulated, airtight container at 39 °C. The pH of the rumen fluid (mean ± SD, 6.94 ± 0.03) was measured using a pH meter (Orion model 260A; Fisher Scientific, Toronto, ON, Canada).

The anaerobic buffer medium (pH 7.07 ± 0.13) was prepared just before rumen sampling using the method described by Goering and Van Soest [19]. The vials were flushed with carbon dioxide, and buffer and rumen fluid were added to each vial at a ratio of 3:1 (60 mL buffer: 20 mL rumen fluid). Each bottle was immediately sealed with a 14-mm butyl rubber stopper plus aluminum crimp cap. All the vials were placed on a rotary shaker platform at 120 revolutions/min in an incubator and kept for 48 h at 39 °C. Blank vials (i.e., buffer and rumen fluid with empty filter bags) were also incubated in three replications. At 3, 6, 12, 18, 24, 36, and 48 h of incubation, the headspace GP was measured with a 23-gauge (0.6 mm) needle attached to a pressure transducer (model PX4200-015GI; Omega Engineering, Inc., Laval, QC, Canada) connected to a visual display unit (Data Track, Christchurch, UK). Pressure values were corrected for background (from buffer and rumen fluid) values by subtracting pressure readings from the blanks. Corrected pressure values were converted to gas volume (mL) estimates using a quadratic equation developed under our laboratory conditions ((4.7047 × gas pressure) + (0.0512 × gas pressure^2^)) [20]. Accumulated gas after 48 h was also expressed relative to the amount of substrate DM degraded.

Immediately after each gas pressure reading, a 20-mL graduated plastic syringe connected to a three-way stopcock was used to sample 15 mL of gas from each vial. The needle was left in the stopper of each vial for approximately 1 min after gas sampling to release the gas. The collected gas was injected into an evacuated 6.8 mL exetainer (Labco Ltd., High Wycombe, England, UK) for gas composition analysis. The CH_4_ concentration was analyzed using a gas chromatograph (Varian 4900; Agilent Technologies Canada Inc., Mississauga, ON, Canada) equipped with a 10 m PoraPLOT U column and thermal conductivity detector as described by Aboagye et al. [18]. After 48 h of incubation, the bags were removed from the vials and washed with cold water until the excess water ran clear. They were dried in an oven at 55 °C for 24 h, and residual DM was weighed to calculate DMD. Methane concentration from the vials was corrected for the blanks and expressed as MP (mL) and MY (mg CH_4_/g DM degraded). The residues in the bags were sequentially analyzed for aNDF to calculate aNDFD.

After 48 h of incubation, 1 mL of the residual liquid in the vials was acidified with 0.2 mL metaphosphoric acid to determine total volatile fatty acid (VFA) concentration, while 1 mL with 0.2 mL of sulfuric acid was used for determination of ammonia concentration. The VFAs were analyzed using gas–liquid chromatography (model 6890; Agilent, Wilmington, DE, USA) with crotonic acid as an internal standard. The ammonia concentration was determined by the salicylate–nitroprusside–hypochlorite method using a segmented flow analyzer (model Astoria2; Astoria Pacific Inc., Clackamas, OR, USA) as described by Fishman [21]. The total VFA and ammonia concentrations were corrected using the blanks after 48 h incubation.

### 2.4. Calculations

Dry-matter degradability and aNDFD were calculated as 100 minus the percentage of DM and aNDF remaining in the bags after 48 h incubation relative to the initial amount incubated. The following equations were used to calculate CH_4_ emission:

Methane production (mL) = Σ(GP_t_ × conc. CH_4t_) − (blank GP_t_ × blank conc. CH_4t_), where t = 3, 6, 12, 18, 24, 36, and 48 h incubation; GP_t_ = gas production (mL); conc. CH_4t_ is the concentration of CH_4_ (%) at time t. Methane yield (mg CH_4_/g DM degraded) = (([(mL CH_4_/1000)/22.4] × 16)/g degraded DM) × 1000, where mL CH_4_ is the accumulated CH_4_ produced after 48 h.

### 2.5. Statistical Analyses

The R version 3.6.1 [22] and “nlme” package by Pinheiro et al. [23] were used to analyze the data and test for lack of independence in the residual and homogeneity of variance. For the batch culture, the three replicates of each WPC variety within a run were averaged prior to statistical analysis. All variables were analyzed using a model that included the fixed effects of location (*n* = 2), crop (*n* = 4), or variety (*n* = 8; 2/crop), and their 2-way interaction and the random effects were run (*n* = 2) and field plot (replicate; *n* = 2). The “emmeans” package by Lenth [24] was used to declare significant differences at *p* < 0.05 and adjusted for comparing means using the Tukey method. Differences among the cereals are shown in tables, whereas crop × location interactions are presented graphically. Where there were variety × location interactions, differences between varieties within species are shown in the tables. The model used was:

Y_ijkl_ = μ + P_i_ + L_j_ + PL_ij_ + β_k_ +R_l_ + e_ijkl_ (1)

where Y_ijkl_ is an observation, μ is the overall mean, P_i_ is the effect of WPC (crop or variety), L_j_ is the effect of location, PL_ik_ is the crop or variety by location interaction, β_k_ is the effect of field plot, R_l_ is the effect of run, and e_ijkl_ is the error term.

The data were combined over locations to appraise the general relationships between MY or MP and chemical composition (starch and aNDF), degradability (DMD and aNDFD), and fermentation end-products (GP and VFA profile). Multiple regression was performed using the “lm” function, and the “step” function was used to identify the best model with the lowest Akaike’s information criterion. The “Hmisc” package [25] was used to identify the correlations between variables measured, while the “corrplot” function from the “corrplot” package [26] was used for graphical representation. Multicollinearity between independent variables was also assessed using the “vif” function, and tolerance level was declared at variance inflation factor < 10. The multiple regression equations, predictors, and correlation coefficients were declared significant at *p* < 0.05, and adjusted R^2^ are reported.

## 3. Results

### 3.1. Whole-Plant DM and Nutrient Concentrations

**Figure 1:**
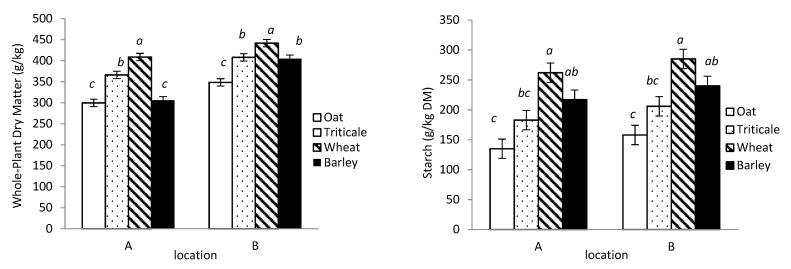

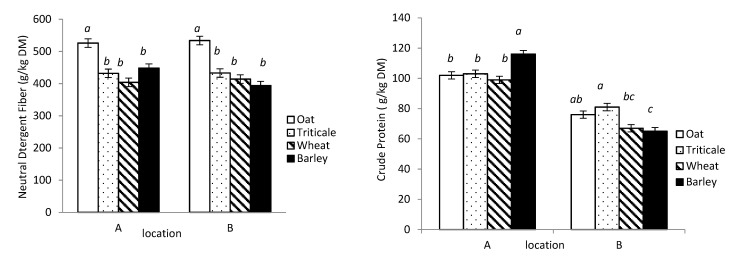
Whole-plant dry matter (**upper left**); starch (**upper right**); neutral detergent fiber (**lower left**); and crude protein (**lower right**) concentrations of oat, triticale, wheat, and barley whole plants grown and harvested at two locations in central Alberta (A = Agriculture and Agri-Food Canada; B = Alberta Agriculture). Means without a common letter differ at *p* < 0.05 (a–c) within the different locations.

The DM concentration of the WPC crops differed depending upon the location (crop × location, *p* < 0.001; Table 1). In both locations, wheat had the greatest DM concentration, followed by triticale, and with oat having the least. The DM concentration of barley was similar to that of oat at Location A, but similar to that of triticale at Location B (*p* < 0.001; Figure 1). The DM concentration of the WPC varieties differed depending on the location, ranging from 287 to 415 g/kg at location A and 335 to 449 g/kg at location B (*p* < 0.001). However, within cereal crops, the DM concentrations of the varieties were similar.

Starch concentration was less for oat than wheat and barley, but similar to that of triticale (*p* < 0.001; Table 1), and was not affected by location. The starch concentration also differed among varieties, with no interaction effect with location, and ranged from 132 to 279 g/kg DM (averaged across locations). Within the same species, the only difference between varieties occurred for triticale, with Sunray greater than Bunker (246 vs. 143 g/kg DM; *p* < 0.001).

The aNDF concentration was greater for oat than the other cereal crops, and was not affected by location (530 vs. 421 g/kg DM; *p* < 0.001; Table 1). There was a variety by location interaction for aNDF concentration, with the values ranging from 402 to 542 g/kg DM at location A and 369 to 538 g/kg DM at location B (*p* < 0.001). Regardless of location, the only difference in aNDF concentration between varieties from the same crop occurred for triticale, with Bunker being 16% greater than Sunray.

There was a crop × location interaction for CP concentration (*p* < 0.001, Figure 1); at location A, where barley had greater CP concentration than the other cereals, whereas at location B, it was similar to wheat, but less than that of oat and triticale. The CP concentrations of all varieties were greater when grown at location A compared with B, ranging from 96 to 127 g/kg DM at location A and from 63 to 83 g/kg DM at location B (*p* < 0.001). The only difference between varieties from the same species occurred for barley at location A, with a 21% greater CP concentration for Champion than CDC Cowboy.

### 3.2. Dry Matter and Fiber Degradabilities

The DMD was greatest for barley, intermediate for wheat and triticale, and least for oat, with no effect of location (*p* < 0.001; Table 2). However, there was a variety × location interaction for DMD, with the only difference between varieties of the same cereal crop occurring in barley, for which Champion was 19% greater than CDC Cowboy at location A, but similar at B.

**Figure 2:**
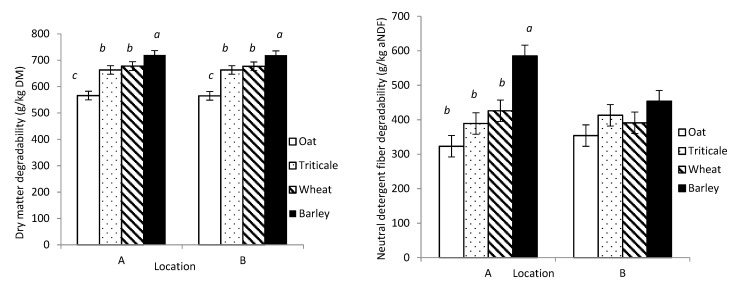

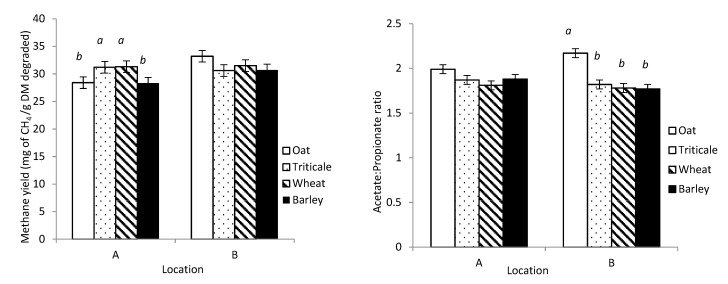
Dry-matter degradability (**upper left**); neutral detergent fiber degradability (**upper right**); methane yield (**lower left**); and acetate:propionate ratio (**lower right**) production of oat, triticale, wheat, and barley whole plants grown and harvested at two locations in central Alberta (A = Agriculture and Agri-Food Canada; B = Alberta Agriculture). Means without a common letter differ at *p* < 0.05 (a,b) within the different locations.

The variations in aNDFD concentration among WPC crops depended on location, with no differences among oat, triticale, and wheat at location B, but greater aNDFD for barley relative to the other cereal crops at location A (crop × location, *p* < 0.04; Figure 2). Similarly, the aNDFD concentration of the varieties was also affected by location, but the variation only occurred in barley varieties at location A, with a 46% greater aNDFD concentration for Champion relative to CDC Cowboy (*p* < 0.001).

### 3.3. Gas Production and Methane Emission

**Figure 3:**
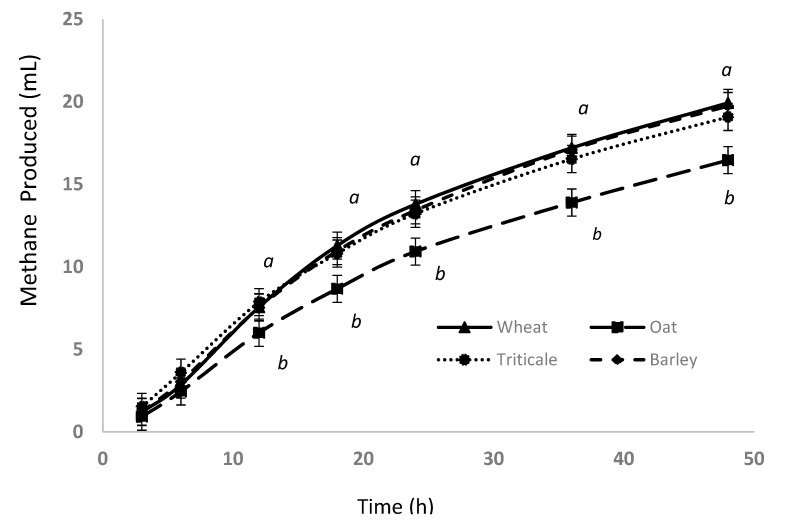
Cumulative methane production of whole-plant cereal crops grown and harvested over two locations in central Alberta. Means without a common letter differ at *p* < 0.05 (a,b).

The cumulative GP after 48 h incubation for the WPC crops or varieties differed depending on location (crop × location, *p* ≤ 0.04; Table 2). The MP did not differ among barley, triticale, and wheat, but it was less for oat compared with the other WPCs. The lower MP of oat was significant between 12 h and 48 h of incubation (6.1 to 16.7 mL versus 7.9 to 19.9 mL; Figure 3). The MP of the WPC crops tended (*p* = 0.06) to be less at location A, with AC Mustang having 24% less MP than CDC Baler at location A (variety × location, *p* < 0.01; Table 2). Similarly for MY, there was a crop × location effect (*p* < 0.01) with similar MY at location B for all the crops (*p* ≥ 0.10), but at location A, MY was less for oat and barley in comparison with triticale and wheat (*p* ≤ 0.04; Figure 2). The MY for WPC varieties was also influenced by location, but the only difference between varieties within a species occurred for oat, where at location A, MY was 16% less for AC Mustang compared with CDC Baler.

### 3.4. Fermentation End-Products

Total VFA production was less for oat compared with the other WPC crops, with no effect of location (*p* ≤ 0.001; Table 3). There was a variety × location interaction for total VFAs, but the differences only occurred among varieties from different crops. The proportion of acetate was greater for oat relative to the other cereal crops (*p* < 0.001), although there was tendency for crop × location interaction (*p* = 0.06) because oat was greater than all crops at a single location. The acetate proportion of the varieties within crop differed for wheat (GP220 > AAC Awesome by 8%) at location A and for barley (CDC Cowboy > Champion by 11%) at location B (*p* < 0.001). There was no difference in propionate proportions among the WPC crops (*p* = 0.50), but there was tendency for a crop × location interaction (*p* = 0.05) because oat was less than triticale and wheat at location B. Similarly, there was a variety × location interaction for propionate proportion; for triticale at location B, Sunray > Bunker by 11%, and for oat at both locations, AC Mustang > CDC Baler by 14% (*p* < 0.001). There was a crop × location interaction for butyrate proportion (*p* = 0.02), with wheat greater than the other cereal crops at location A and barley and wheat greater than oat and triticale at location B. Location also affected the proportion of butyrate for the varieties, with inconsistent differences between varieties of the same species occurring for wheat (AAC Awesome > GP220 by 27%) at location A and for barley (Champion > CDC Cowboy by 27%) at location B (*p* < 0.001). The acetate:propionate ratio for the cereal crops and varieties followed the trend for acetate and propionate proportions (crop × location, *p* = 0.05; Figure 2). There were no differences among the crops at location A, but at location B, the acetate:propionate ratio was greater for oat than the other crops.

The ammonia concentration was less for oat compared with the average of barley, triticale, and wheat, irrespective of location (5.5 vs. 6.9 mM; *p* < 0.001; Table 3). However, ammonia concentration was less for crops grown at location B relative to location A (5.6 vs. 5.7 mM; *p* < 0.001). There was a variety × location interaction for ammonia concentration (*p* = 0.045); however, the differences only occurred for varieties of different species.

### 3.5. Relationships and Models

**Figure 4:**
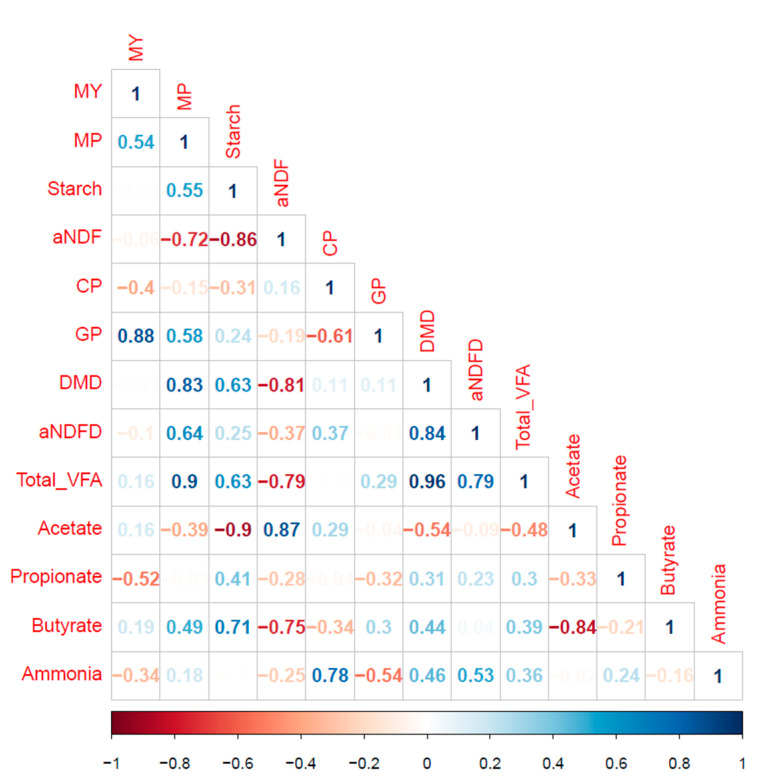
A correlogram showing the correlation between chemical composition, degradation, and fermentation end-products over location and whole-plant cereal varieties grown and harvested in central Alberta. Positive and negative correlation coefficients are displayed in blue and red scale, respectively (0.5 to 1 or −1 to −0.5; *p* < 0.05). All chemical composition in g/kg DM; ammonia is m*M*; CP is crude protein (g/kg DM); DMD is in vitro dry-matter degradability (g/kg DM); GP is cumulative gas produced after 48 h (mL/g DM degraded); MP is methane production (mL); MY is methane yield (mg CH_4_/g DM degraded); aNDF is neutral detergent fiber-inclusive ash (g/kg DM); aNDFD is in vitro neutral detergent fiber degradability (g/kg aNDF); Total_VFA is total volatile fatty acid (mM); VFA profile (acetate, propionate and butyrate) in mol/100 mol.

When examined over varieties and locations for the different expressions of CH_4_ emission, MP was inversely correlated to aNDF concentration (*r* = −0.72) and positively to starch (*r* = 0.55), DMD (*r* = 0.83), aNDFD (*r* = 0.64), GP after 48 h (*r* = 0.58), and total VFA production (*r* = 0.90; *p* ≤ 0.03; Table 4). The MY was correlated positively with GP after 48 h (*r* = 0.88) and negatively with propionate (*r* = −0.52, *p* ≤ 0.038). Some of the variables were correlated (Figure 4).

Multiple regression analysis showed that chemical composition alone explained 45% of the variation in MP, while the combination of chemical composition and nutrient degradabilities explained 66% of the variation (*p* ≤ 0.02; Table 5). However, the best-fit prediction for MP was DMD combined with GP at 48 h, explaining 92% of the variation (*p* < 0.001). In contrast, chemical composition (starch, aNDF, and CP concentrations) of the WPC alone or together with nutrient degradabilities (DMD and aNDFD) failed to explain the variation in MY (R^2^ ≤ 0.04; *p* ≥ 0.35). The variation in MY was mostly explained by the combined effects of aNDFD concentration, GP after 48 h, total VFA production, proportions of acetate and butyrate, and ammonia concentration (R^2^ = 0.88; *p* < 0.001).

## 4. Discussion

Small-grain cereal forages (barley, oat, triticale, and wheat) are cool-season crops and therefore tolerate the growing season conditions of many northern latitudes [27]. The emergence of superior varieties of cereal species has led to high biomass yields (7 to 16 t DM/ha; [28]) of WPC forages, with superior nutritional quality [8,9,10]. While economic production of forages must optimize nutritional quality and animal productivity, there is also a need to consider effects on enteric MP. Generally, an increase in the non-fiber fraction of feed decreases MP, while increased DMD increases MP due to a greater supply of fermentable substrate [29,30,31]. Our study confirms that the variable concentrations of starch and fiber in WPCs affected their in vitro degradability and fermentation profiles, and these variations affected MP and MY.

Many studies that have evaluated forage quality used samples obtained from a single growing site [10,28]. Including two field locations in the present study led to a number of significant location, location × crop, and location × variety effects despite the locations being in the same geographical area of Lacombe, Alberta. Location effects reflected differences in agronomic conditions, including precipitation, fertilization, microclimate, and management, and led to increased variability in the quality variables measured, as intended. In addition, two varieties of each cereal were preselected to further increase the range in chemical composition and degradability of each cereal crop. It is also important to note that in the present study, the forages were not ensiled, and thus the quality data represent pre-ensiling nutritional quality. However, chemical composition and degradability are not expected to change markedly during the ensiling process [32].

### 4.1. Whole-Plant DM and Nutrient Concentrations of WPCs

Based on DM concentration, the crops at location B were at a later stage of development at harvest compared with those at location A. However, at both locations, the DM concentrations of the WPC were generally within the range recommended for silage production to reduce effluent and oxidative loses (300 to 400 g/kg; [33]), with the exception of wheat (426 g/kg), which was slightly greater. These results indicate a faster drying rate for wheat relative to the other crops as grain-filling advanced. Similarly, Rosser et al. [28] reported that DM concentration of wheat was greater than that of barley and oat at the late-milk and hard-dough stages. At later growth stages, wheat can mature rapidly and become too dry to ensile successfully, which may negatively affect its palatability and nutritional value. In contrast to our results, Lyu et al. [34] reported that DM concentration of triticale was greater than that of barley when harvested at a similar stage of maturity.

As WPCs mature, starch concentration increases, and aNDF concentration decreases correspondingly due to a dilution effect of grain development [35]. In the present study, wheat, barley, and triticale were all harvested at the soft-dough stage, whereas oat was harvested at the milk stage. The earlier stage of maturity of oat at harvest accounts for its lower concentration of starch compared with barley and wheat. Although the starch concentration of oat did not differ from the mean for triticale, this was mainly because Bunker triticale had a low starch concentration compared to that of Sunray triticale. The low starch concentration of oat was insufficient to dilute the fiber from the stalk as the plant matured, leading to its greater aNDF concentration compared with the other crops. Greater aNDF concentration of whole-plant oat relative to triticale and barley [36] and wheat [35] was previously reported.

The CP concentration of WPCs decreases as maturity progresses [36,37]. The range of CP concentrations of the WPC crops and varieties in the present study was influenced by location, likely reflecting differences in the nitrogen fertilization of the fields. The CP concentrations ranged from 63 to 127 g/kg DM and were comparable to values for WPCs grown and harvested in western Canada at similar stages of maturity (93 to 125 g/kg DM; [28,34]).

### 4.2. In Vitro DMD, aNDFD, and Fermentation End-Products

Variations in starch, aNDF, and aNDFD concentrations led to variations in DMD among the WPC crops (566 to 719 g/kg DM) and varieties (556 to 785 g/kg DM). These differences are comparable to an earlier study [38] that showed that, at the late-milk stage of maturity, the DMD of oat (574 g/kg DM) was 13.9%, 23.0%, and 24.4% less than that of barley, wheat, and triticale, respectively. The decreased DMD of oat in the present study was mainly due to its greater aNDF concentration, which was less degradable relative to the other crops. Therefore, as expected, the total production of ruminal VFA, which forms a major metabolic component of the digestible energy requirement of cattle (75% to 80%; [39]), was 22.6% less for oat compared with the other cereal forages.

The fiber in barley was highly degradable (519 g/kg aNDF), especially for the Champion variety (695 kg/kg aNDF), leading to a 6.8% greater DMD compared with wheat and triticale. In other in vitro studies, the barley variety CDC Cowboy harvested as WPC or ensiled at the mid-dough maturity stage contained less starch and greater aNDF concentrations than the barley variety Xena; however, the aNDFD of CDC Cowboy was greater [40] or did not differ [10,41] from that of Xena (a hulless variety low in aNDF concentration). CDC Cowboy is a barley variety that was specifically developed for forage rather than grain production, which accounts for its relatively low starch concentration.

Harvesting WPCs at the late-milk to late-dough stages for silage production has been shown to maximize total digestible nutrient concentration [42], which is a function of nutrient composition and digestibility. Greater starch and lower aNDF concentrations, combined with greater degradability, increases total digestible nutrient concentration and nutritional value of forages. With ensiled cereal forages harvested at the same stages of maturity as used in the present study (i.e., milk stage for oat and soft-dough stage for barley and triticale), barley silage had 8.8% greater total-tract DM digestibility in sheep, and resulted in 18.5% greater average daily gain of growing heifers when compared to the average of oat and triticale silages [32]. Thus, overall, the greater DMD and aNDFD of barley compared with the other WPC in the present study would be expected to maximize animal performance.

### 4.3. Gas Production and CH_4_ Emission

Differences in chemical composition and degradabilities among the WPCs led to differences in CH_4_ emission potential, expressed as MP (mL) and MY (mg CH_4_/g DM degraded). The MP of the WPC forages corresponded positively to starch concentration (*r* = 0.55), degradability (DMD, *r* = 83; aNDFD, *r* = 0.64), GP (*r* = 0.58), and total VFA (*r* = 0.90), and inversely to aNDF (*r* = −0.72) concentration. Thus, it is not surprising that oat, with the least starch and greatest aNDF concentrations, and lowest degradabilities, total VFA production, and GP, had the least accumulated MP from 12 h to 48 h. Moreover, some types of antioxidants, such as avenanthramides, are present in oat and not in other cereal grains [43], which may also lower the MP of oat relative to other WPC crops. The increase in R^2^ from 0.45 to 0.66 by including degradability (DMD and aNDFD) in the prediction equations of MP, compared with chemical composition alone, indicates the importance of rumen degradability of carbohydrates. Similarly, it was previously reported for dairy cows that predictions of MP (g/day) were improved by including digestible aNDF concentration in addition to aNDF concentration [44]. In the present study, the best-fit model for MP combined DMD and GP (R^2^ = 0.92); a unit increase in DMD increased in vitro MP by 0.24 mL while a unit increase in GP increased it by 0.42 mL. Accounting for rumen fermentation variables did not further improve the prediction of MP, likely because DMD was highly positively correlated to total VFA and negatively correlated to acetate proportion. Ramin and Huhtanen [45] reported that the MP of cows tended to increase with increasing diet organic matter digestibility, while Storlien et al. [46] showed that accounting for total digested nutrients improved model predictions of MP for dairy cows.

When CH_4_ potential was expressed as MY, the chemical composition variables did not explain a significant portion of the variability (R^2^ = 0.04; *p* = 0.35), and predictions were not further improved by incorporating degradability variables (R^2^ = −0.045; *p* = 0.35). Similarly for whole-plant corn hybrids, previous research showed that chemical composition variables failed to explain in vitro MY variation [18]. In the present study, the best-fit equation for MY accounted for negative effects of NDFD and positive effects of GP, acetate, butyrate, and ammonia (R^2^ = 0.88). Thus, once CH_4_ emissions were adjusted for differences in DMD, by using MY accounting for the fermentability of the fiber fraction and the fermentation profile further explained the observed variability in CH_4_. The importance of acetate in the prediction model can be attributed to its high positive correlation with aNDF concentration (*r* = 0.87), and negative correlation with starch concentration (*r* = −0.90). Although propionate was negatively correlated with MY (*r* = −0.52), it surprisingly did not factor into the prediction models for MY due to collinearity among variables (acetate and butyrate). Production of propionate uses reducing equivalents and stoichiometrically lowers hydrogen available for CH_4_ formation [7].

The multiple regression approach used in the present study highlights the inter-relationships among variables and the complexity of predicting CH_4_ potential of forages. The results indicate that increased starch concentration dilutes aNDF concentration of WPC, and in turn increases DMD and fermentability (total GP and total VFA), and consequently MP is increased. When adjusted for differences in DMD, CH_4_ from WPC is mainly influenced by NDFD and fermentability (GP and fermentation end-products).

The study indicates that growing and harvesting WPC forages to maximize DMD and animal performance will likely also increase daily MP. However, increased animal productivity leads to decreased emission intensity (CH_4_/animal product) [47]. Selecting varieties with higher fiber degradability will help reduce MY, in addition to improving animal performance.

Finally, it should be noted that the use of ground samples, a 48-h incubation time, and buffered media in the in vitro study would be expected to maximize degradability, fermentation, and CH_4_ emission. Thus, the results should be considered as potential maximums. Use of ground samples removes the physical barriers to degradability, unlike in animal studies where mastication of feed is necessary to break down particles enabling access to rumen microbes. Additionally, a 48-h incubation time may exceed the retention time of forage particles in the rumen of some animals (e.g., dairy cows), and the low rumen pH of some animals can limit fiber degradability. Therefore, in vitro rumen degradability is not equivalent to total-tract in vivo digestibility, although these variables are correlated. Rustas et al. [48] reported for WPC harvested at the milk and dough stages a strong relationship (R^2^ = 0.75; *p* < 0.001) between in vitro organic matter digestibility after 96 h of incubation and in vivo organic matter digestibility using dairy animals. Similarly, Oba and Allen [49], using 13 data sets from seven in vitro studies, reported that an increase in aNDFD after 24, 30, and 48-h incubations was associated with an increase in dairy animal performance.

## 5. Conclusions

Barley, oat, triticale, and wheat grown in two locations and harvested as whole-plant forage varied in nutrient composition, which affected rumen degradability, fermentation, and CH_4_ emission potential. When averaged over varieties and locations, MP from the cereal forages was mainly influenced by DMD and fermentation (i.e., GP). When CH_4_ was expressed relative to DM degraded (i.e., MY), differences were due to fiber degradation and fermentation end-products (GP, acetate, butyrate, and ammonia). The MP from oat was less than that of the other cereal forages due to its lower DMD resulting from greater aNDF and lower starch concentrations. The MY was less for barley relative to wheat or triticale because of its greater aNDFD. These results indicate that the use of oat as a forage may decrease CH_4_ emission of ruminants, but animal performance would be expected to be negatively affected due to its lower DMD. In contrast, use of barley forage may reduce CH_4_ emissions with no negative effects on animal performance. Further in vivo studies with cereal forages grown and harvested in a number of locations are needed to confirm the relationships reported in the present study.

## Figures and Tables

**Table 1:** Whole-plant dry matter (DM), starch, neutral detergent fiber (aNDF), and crude protein (CP) concentrations of whole-plant cereal crops grown and harvested at two locations (A, B) in central Alberta.

Item	DM (g/kg) ^1^		Starch (g/kg DM) ^1^		aNDF (g/kg DM) ^1^		CP (g/kg DM) ^1^	
	A	B	A	B	A	B	A	B
Whole-plant crop								
Barley	355c		229ab		421b		91a	
CDC Cowboy	325	407	199	212	450	418	105b	63
Champion	287	399	205	298	447	369	127a	68
Oat	325d		147c		530a		89ab	
AC Mustang	296	363	155	167	542	538	96	74
CDC Baler	306	335	141	124	511	531	108	78
Triticale	388b		194bc		433b		92a	
Bunker	369	403	138	147	463	467	102	83
Sunray	373	408	218	275	402	400	104	78
Wheat	426a		274a		409b		83b	
AAC Awesome	399	440	277	281	403	410	99	67
GP220	415	449	264	273	404	417	100	67
SEM ^2^								
Crop	6.3		14.5		9.3		1.9	
Location	5.3		10.2		6.6		1.3	
Crop × location	8.8		16.2		13.2		2.4	
*p*-value								
Crop	<0.001		<0.001		<0.001		0.01	
Location	<0.001		0.14		0.34		<0.001	
Crop × location	<0.001		0.53		0.07		<0.001	
SEM ^2^								
Variety	7.4		11.9		9.1		2.1	
Location	5.2		5.9		5.3		1.1	
Variety × location	9.5		16.8		12.5		2.8	
*p*-value								
Variety	<0.001		<0.001		<0.001		<0.001	
Location	<0.001		0.02		0.16		<0.001	
Variety × location	<0.001		0.10		0.02		<0.001	

^1^ Different lowercase letters following means differ at *p* < 0.05 (a–c) for crop effect, and variety within species based on variety × location interaction; *n* = 4 for each variety within location (A = Agriculture and Agri-Food Canada; B = Alberta Agriculture). ^2^ Standard error of mean.

**Table 2:** In vitro dry-matter degradability (DMD), neutral detergent fiber degradability (aNDFD), accumulated gas produced (GP), methane yield (MY), and methane production (MP) after 48 h for whole-plant cereal crops grown and harvested at two locations (A, B) in central Alberta.

Item	DMD ^1^ (g/kg DM)		aNDFD ^1^ (g/kg aNDF)		GP ^1^ (mL/g DM Degraded)		MP ^1^ (mL)		MY ^1^ (mg/g DM Degraded)	
	A	B	A	B	A	B	A	B	A	B
Whole-plant crop										
Barley	719a		519a		355		20a		30	
CDC Cowboy	660b	718	475b	481	338	376	17	21	28	31
Champion	785a	715	695a	426	341	363	21	20	28	30
Oat	566c		339b		359		16b		31	
AC Mustang	529	583	283	393	325	377	13b	18	26b	33
CDC Baler	594	556	364	315	352	379	17a	18	31a	34
Triticale	663b		401b		354		19a		31	
Bunker	637	642	370	405	339	351	18	19	31	31
Sunray	680	693	409	421	367	358	20	19	32	30
Wheat	677b		409b		363		20a		31	
AAC Awesome	658	676	358	384	351	363	18	20	30	31
GP220	710	666	493	399	370	368	22	20	33	32
SEM ^2^										
Crop	15.5		22.0		4.8		0.9		1.0	
Location	13.3		14.3		3.7		0.8		0.9	
Crop × location	16.4		31.1		6.4		1.0		1.1	
*p*-value										
Crop	<0.001		<0.001		0.41		<0.001		0.08	
Location	0.97		0.21		<0.001		0.06		<0.01	
Crop × location	0.88		0.04		0.01		0.10		<0.01	
SEM ^2^										
Variety	16.0		24.5		4.9		1.0		1.0	
Location	13.1		13.4		3.6		0.8		0.8	
Variety × location	20.5		37.2		8.3		1.1		1.2	
*p*-value										
Variety	<0.001		<0.001		0.08		<0.001		<0.01	
Location	0.92		0.17		<0.01		0.02		<0.01	
Variety × location	0.003		<0.001		0.04		0.01		<0.01	

^1^ Different lowercase letters following means differ at *p* < 0.05 (a–c) for crop effect, and variety within species based on variety × location interaction; *n* = 4 for each variety within location (A = Agriculture and Agri-Food Canada; B = Alberta Agriculture). ^2^ Standard error of mean.

**Table 3:** In vitro total volatile fatty acid (VFA) concentration and individual VFA proportions of whole-plant cereal crops grown and harvested at two locations (A, B) in central Alberta.

Item	Total VFA ^1,2^ (mM)		Acetate (A)		Propionate (P)		Butyrate		A:P ^1^		Ammonia ^1,2^ (m*M*)	
	A	B	A	B	A	B	A	B	A	B	A	B
Whole-plant crop												
Barley	81ab		52b		28		15ab		1.8b		6.9ab	
CDC Cowboy	75	83	52	52a	28ab	29	15	15b	1.9	1.8	7.5	5.3
Champion	88	79	54	47b	29ab	27	13	19a	1.9	1.7	9.1	5.8
Oat	64c		56a		27		12c		2.1a		5.5c	
AC Mustang	56	67	55	56	30a	28a	11	13	1.8b	2.0	6.1	4.9
CDC Baler	66	66	57	57	26b	25b	12	13	2.2a	2.3	6.8	4.2
Triticale	76b		52b		28		14bc		1.8b		7.5a	
Bunker	72	70	54	54	28	27b	13	15	1.9	2.0a	8.8	6.3
Sunray	79	81	52	50	28	30a	15	14	1.8	1.6b	8.1	6.9
Wheat	78b		50b		28		16ab		1.8b		6.3b	
AAC Awesome	72	80	48b	50	27	30	19a	15	1.7	1.7	6.3	4.9
GP220	82	77	52a	51	28	28	15b	16	1.9	1.8	7.6	6.5
SEM ^3^												
Crop	1.6		0.9		0.5		0.5		0.03		0.23	
Location	1.3		0.9		0.4		0.5		0.03		0.16	
Crop × location	2.6		1.0		0.7		0.8		0.05		0.33	
*p*-value												
Crop	<0.001		<0.001		0.29		<0.001		<0.001		<0.001	
Location	0.37		0.19		0.85		0.05		0.96		<0.001	
Crop × location	0.67		0.06		0.05		0.02		0.05		0.15	
SEM ^3^												
Variety	2.0		1.0		0.5		0.6		0.03		0.27	
Location	1.2		1.2		0.4		0.4		0.02		0.14	
Variety × location	3.3		1.4		0.7		0.9		0.05		0.39	
*p*-value												
Variety	<0.001		<0.001		<0.001		<0.001		<0.001		<0.001	
Location	0.55		0.09		0.881		0.02		0.72		<0.001	
Variety × location	0.04		<0.001		0.003		<0.001		0.002		0.045	

^1^ Different lowercase letters following means differ at *p* < 0.05 (a–c) for crop effect, and variety within species based on variety × location interaction; *n* = 4 for each variety within location (A = Agriculture and Agri-Food Canada; B = Alberta Agriculture). ^2^ Total volatile fatty acid and ammonia corrected from blank (buffer and rumen fluid with empty filter bags) after 48 h incubation period. ^3^ Standard error of means.

**Table 4:** Pearson **c**orrelation coefficient and significance (*p-*values) in parentheses between methane emissions and chemical composition, degradation, and fermentation end-products examined over two locations and two varieties of each cereal crop (barley, oat, triticale, and wheat) grown and harvested in central Alberta.

Item	MP ^1^ (mL)	MY ^2^ (mg CH_4_/g DM Degraded)
Starch (g/kg DM)	0.55 (0.03)	−0.01 (0.96)
aNDF ^3^ (g/kg DM)	−0.72 (0.002)	−0.06 (0.84)
CP ^4^ (g/kg DM)	−0.15 (0.59)	−0.40 (0.13)
DMD ^5^ (g/kg DM)	0.83 (<0.001)	−0.01 (0.95)
aNDFD ^6^ (g/kg aNDF)	0.64 (0.001)	−0.10 (0.70)
GP ^7^ (mL/g DM degraded)	0.58 (0.02)	0.88 (<0.001)
Ammonia (mM)	0.18 (0.21)	−0.34 (0.21)
Total VFA ^8^ (mM)	0.90 (<0.001)	0.16 (0.55)
Acetate (mol/100 mol)	−0.39 (0.14)	0.16 (0.55)
Propionate (mol/100 mol)	−0.03 (0.92)	−0.52 (0.038)
Butyrate (mol/100 mol)	0.49 (0.052)	0.19 (0.48)

^1^ MP is methane production; ^2^ MY is methane yield; ^3^ aNDF is neutral detergent fiber-inclusive ash. ^4^ CP is crude protein. ^5^ DMD is in vitro dry-matter degradability. ^6^ aNDFD is in vitro neutral detergent fiber degradability. ^7^ GP is cumulative gas produced after 48 h. ^8^ Total VFA is total volatile fatty acid.

**Table 5:** Prediction equations for methane based on chemical composition, degradation, and fermentation end-products over two locations and two varieties of each cereal crop (barley, oat, triticale, and wheat) grown and harvested in central Alberta.

Item	Model Equation ^1^	Adjusted R^2^ (*p*-Value) ^2^
Methane production (mL)		
Chemical composition	MP = 30.91 − 0.27 aNDF − 0.10 CP	0.45 (0.02)
Chemical composition and degradability	MP = 24.59 − 0.11 starch − 0.22 aNDF − 0.36 CP + 0.09 DMD + 0.09 aNDFD	0.66 (0.01)
Chemical composition, degradability, gas and VFA ^3^	MP = −20.14 + 0.24 DMD + 0.06 GP.48h	0.92 (<0.001)
Methane yield (mg CH_4_/g DM degraded)		
Chemical composition	MY = 46.28 − 0.18 starch − 0.16 aNDF − 0.52 CP	0.04 (0.35)
Chemical composition and degradability	MY = 59.47 − 0.20 starch − 0.28 aNDF − 0.59 CP − 0.17 DMD + 0.09 aNDFD	−0.045 (0.53)
Chemical composition degradability, gas and VFA ^3^	MY = −38.94 − 0.05 aNDFD + 0.12 GP.48h + 0.36 acetate + 0.38 butyrate + 0.57 ammonia	0.88 (<0.001)

^1^ Multiple regression analysis was used to develop methane prediction equations; acetate is molar proportion (mol/100 mol); butyrate is molar proportion (mol/100 mol); ammonia is ruminal ammonia concentration (mM); CP is crude protein (g/kg DM); DMD is in vitro dry-matter degradability (g/kg DM); GP.48h is cumulative gas produced after 48 h (mL/g DM degraded); MP is methane production (mL); MY is methane yield (mg CH_4_/g DM degraded); aNDF is neutral detergent fiber-inclusive ash (g/kg DM); aNDFD is in vitro neutral detergent fiber degradability (g/kg aNDF); propionate is molar proportion (mol/100 mol); total VFA is total volatile fatty acid (mM); ^2^ *p-*values in parentheses. ^3^ The “step” function in R software was used to identify models that best explained variations in MY and MP when all predictors were considered.

## Data Availability

Data available upon request.
